# The Reliability and Validity of the Perceived Dietary Adherence Questionnaire for People with Type 2 Diabetes

**DOI:** 10.3390/nu7075231

**Published:** 2015-07-07

**Authors:** Ghada Asaad, Maryam Sadegian, Rita Lau, Yunke Xu, Diana C. Soria-Contreras, Rhonda C. Bell, Catherine B. Chan

**Affiliations:** 1 Department of Agricultural, Food and Nutritional Science, University of Alberta, Edmonton, AB T6G 2R3, Canada; E-Mails: gasaad@ualberta.ca (G.A.); sadegian@ualberta.ca (M.S.); yikkwan@ualberta.ca (R.L.); yunke2@ualberta.ca (Y.X.); soria@ualberta.ca (D.C.S.-C.); rhonda.bell@ualberta.ca (R.C.B.); 2 Department of Physiology, University of Alberta, Edmonton, AB T6G 2R3, Canada

**Keywords:** dietary guidelines, nutritional assessment, type 2 diabetes

## Abstract

Nutrition therapy is essential for diabetes treatment, and assessment of dietary intake can be time consuming. The purpose of this study was to develop a reliable and valid instrument to measure diabetic patients’ adherence to Canadian diabetes nutrition recommendations. Specific information derived from three, repeated 24-h dietary recalls of 64 type 2 diabetic patients, aged 59.2 ± 9.7 years, was correlated with a total score and individual items of the Perceived Dietary Adherence Questionnaire (PDAQ). Test-retest reliability was completed by 27 type 2 diabetic patients, aged 62.8 ± 8.4 years. The correlation coefficients for PDAQ items *versus* 24-h recalls ranged from 0.46 to 0.11. The intra-class correlation (0.78) was acceptable, indicating good reliability. The results suggest that PDAQ is a valid and reliable measure of diabetes nutrition recommendations. Because it is quick to administer and score, it may be useful as a screening tool in research and as a clinical tool to monitor dietary adherence.

## 1. Introduction

There has been an increase in the incidence of diabetes worldwide. Over 347 million individuals have diabetes and it is estimated that by the year 2030, 552 million people will be living with diabetes [1]. In Canada, 2.4 million people had diabetes in 2009 and by 2019 the number is expected to reach 3.7 million [2]. The economic burden of diabetes in Canada is estimated to rise from $6.3 billion annually in 2000 and to $16.9 billion in 2020 [3]. Nutrition therapy is a crucial part of type 2 diabetes treatment and self-management. It has been well documented that improving dietary intake can reduce glycated hemoglobin (A1c) [4,5], improve clinical outcomes, and mediate weight loss [5,6]. The Canadian Diabetes Association (CDA) [7] recommends that diabetic patients to follow Eating Well with Canada’s Food Guide (CFG) [8] in order to meet their nutrition requirements. Additional recommendations include limiting saturated fat and restricting added sucrose plus fructose to 10% of total energy while increasing consumption of low glycemic index foods, high-fiber foods, monounsaturated fats and foods rich in *n*-3 fatty acids [7].

While clinical outcomes such as A1c and blood pressure can easily be monitored by the medical team treating the diabetic patient, assessing dietary intake and creating a longitudinal record of dietary intake is not as practical [9]. However, being able to monitor a health outcome and provide timely feedback to the patient may help in long-term adherence to dietary goals [10]. Dietary intake is usually assessed by 24-h recalls, food frequency questionnaires (FFQ) and food records. These instruments require administration and analysis by a skilled health care professional [11]. Therefore, these instruments are not suitable for quick assessment by health care providers. They may also impose a significant patient burden [9,10,11]. Furthermore, these instruments are not specific for diabetes diet recommendations; therefore, the questionnaires may not be sensitive enough to assess how well a patient is adhering to a prescribed dietary pattern. For example, our previous study found no significant change in the Healthy Eating Index score calculated from 3-day food records after 12 weeks of following a menu plan for diabetes, despite significantly lower body mass index, waist circumference and A1c [12]. A shortcoming of the Healthy Eating Index is that it was not developed for people with diabetes; it incorporates general food guide serving recommendations but not specific diabetes recommendations. Few studies have developed a questionnaire to measure the adherence to disease-relevant guidelines [13,14] or specific diets [15,16] and there is no short questionnaire to measure a combination of the adherence to CFG [8] and CDA recommendations [7] in individuals with type 2 diabetes. Therefore, the Perceived Dietary Adherence Questionnaire (PDAQ) was developed to measure diabetic patients’ perceptions of their dietary adherence. The present study aimed to measure the reliability of PDAQ and its validity relative to three repeated 24-h dietary recalls.

## 2. Experimental Section

### 2.1. Subjects

Data from the Physical Activity and Nutrition for Diabetes in Alberta (PANDA) intervention study (ClinicalTrials.gov registration NCT01625507) were used to test internal consistency and validity. Briefly, 73 participants were enrolled in the dietary intervention (Cohort I). Participants were recruited through a variety of avenues including posters, word-of-mouth, contact via a list of potential participants maintained by the Alberta Diabetes Institute and via an article about the project in a local newspaper. The inclusion criteria were: people diagnosed with type 2 diabetes and able to read and speak English. The exclusion criteria were: having severe gastrointestinal issues, type 1 diabetes or kidney disease. Anthropometric measures (height, weight, waist circumference), A1c, blood pressure, serum lipids, 24-h recall repeated on three successive days, and PDAQ were obtained at baseline and three months. Subsequently, additional type 2 diabetes patients (*n* = 27, Cohort II) were recruited through poster and database of the Alberta Diabetes Institute to measure the test and retest reliability of the PDAQ with a one-week interval. The inclusion and the exclusion criteria were the same as for the intervention study. The University of Alberta Human Research Ethics Board approved both studies. Written informed consent was obtained from all participants.

### 2.2. Perceived Dietary Adherence Questionnaire

The PDAQ was adapted from the Summary of Diabetes Self-care Activities measure [17]. The questionnaire was modified according to CFG [8] and the CDA Nutrition Therapy recommendations in place in the 2008 Clinical Practice guidelines [18]. To test item clarity, four experts were involved in reviewing the questionnaire items and the PDAQ was pre-tested on 10 non-diabetic volunteers. Questions raised by the pre-test cohort were addressed prior to using PDAQ in a research cohort.

The questionnaire consists of a total of nine questions structured to cover the CDA Nutrition Therapy guidelines [18] with reference to following CFG [8]: overall adherence to CFG, recommended fruits and vegetables servings, consumption of low glycemic index carbohydrate-containing foods, high sugar foods, high fiber foods, *n*-3 fatty acids, healthy (monounsaturated) oils, and high fat foods. One item addresses appropriate carbohydrate spacing. The response is based on a seven-point Likert scale to answer the question phrased as “On how many of the last 7 days did you…?” (Table 1). Higher scores reflect higher adherence except for items 4 and 9, which reflect unhealthy choices (foods high in sugar or fat). For these items, higher scores reflect lower adherence, therefore, for computing a total PDAQ score, the scores for these items were inverted. Although based on a weekly timeframe, it was anticipated that the PDAQ would reflect usual dietary patterns based on knowledge that most people consume similar foods from week to week [19].

**Table 1:** Perceived Dietary Adherence Questionnaire (PDAQ).

Item	Response *
1. On how many of the last SEVEN DAYS have you followed a healthful eating plan such as Eating Well with Canada’s Food Guide with appropriate serving sizes?	0 1 2 3 4 5 6 7
2. On how many of the last SEVEN DAYS did you eat the number of fruit and vegetable servings you are supposed to eat based on Canada’s Food Guide?	0 1 2 3 4 5 6 7
3. On how many of the last SEVEN DAYS did you eat carbohydrate-containing foods with a low Glycemic Index? (Example: dried beans, lentils, barley, pasta, low fat dairy products)	0 1 2 3 4 5 6 7
4. On how many of the last SEVEN DAYS did you eat foods high in sugar, such as cakes, cookies, desserts, candies, *etc.*?	0 1 2 3 4 5 6 7
5. On how many of the last SEVEN DAYS did you eat foods high in fibre such as oatmeal, high fiber cereals, whole-grain breads?	0 1 2 3 4 5 6 7
6. On how many of the last SEVEN DAYS did you space carbohydrates evenly throughout the day?	0 1 2 3 4 5 6 7
7. On how many of the last SEVEN DAYS did you eat fish or other foods high in omega-3 fats?	0 1 2 3 4 5 6 7
8. On how many of the last SEVEN DAYS did you eat foods that contained or was prepared with canola, walnut, olive, or flax oils?	0 1 2 3 4 5 6 7
9. On how many of the last SEVEN DAYS did you eat foods high in fat (such as high fat dairy products, fatty meat, fried foods or deep fried foods)?	0 1 2 3 4 5 6 7

* Scoring: to obtain the total PDAQ score, the responses for items 4 and 9 were first inverted, e.g., a score of 7 becomes 0, then add all of the responses together. The maximum score was 63.

### 2.3. Assessment of Dietary Intake

Baseline dietary intake of Cohort I was measured by three 24-h dietary recalls (2 weekdays and 1 weekend day) using an internet-based questionnaire (WebSpan), which has been shown to reduce assessment error and bias [20]. Daily records were screened for duplicate entries for a single food item. Participants were excluded from the analysis if they did not completed three 24-h dietary recalls or implausible total energy values were reported (outside the range of 500–3500 kcal/day for women and 800–4000 kcal/day for men) [21]. The average daily total energy, macronutrient intake and intake from the four food groups described in CFG was obtained from WebSpan based on the 2001b Canadian Nutrient File database [22], and used for analysis. To calculate the Healthy Eating Index (HEI) [23] the food items reported by the participants and macronutrient analysis from WebSpan were used. Glycemic index (GI) score was calculated by following formula (daily GI = GL/net carbohydrate × 100). GI values were obtained from two databases [24,25]. Carbohydrate spacing was measured by calculating grams of carbohydrate consumed at each meal and snack [26], then giving a score from 1–6, where 1 represented poor spacing of carbohydrates (all in one meal) and six represented excellent spacing of carbohydrates (at least 15 g per meal and snack). PDAQ takes approximately 5 min for participants to complete and one minute to calculate the score, which was based on a maximum of 7 for each item (with the items for consumption of foods high in sugar and fat inversely scored), for a total maximum score of 63.

### 2.4. Statistical Methods

Statistical analyses were performed using SPSS version 22.0 (SPSS Inc., Chicago, IL, USA), Statistical significance was set at *p* < 0.05. Descriptive statistics were used to summarize demographic data. The mean ± SD was calculated for continuous variables, and percentage for categorical variables. Comparison of demographic characteristics between the first cohort (used to test validity) and the second cohort (used to do test-retest reliability) was assessed by Chi square and unpaired *t*-tests as appropriate. Spearman rank-order correlation coefficients were calculated between PDAQ questions to determine if the perceived adherence to CFG question score (Question (1)) correlated with the scores of Questions (2) through (9).

Validation: After screening the food intake data for implausible dietary intake or incomplete three 24-h dietary recalls, nine participants were removed (*n* = 64). Normality of nutrient intake distributions was checked statistically. If the normality assumption failed, data were log10-transformed. The questions of the PDAQ were individually correlated with specific information derived from the three 24-h dietary recalls (*i.e*., mean servings of food groups, nutrient intakes, glycemic index). Specifically, the question related to CFG was correlated with the mean number of servings of the four food groups. The question related to vegetables and fruits consumption was correlated with the mean servings of vegetables and fruits. The question related to consumption of foods with low glycemic index was correlated with the mean glycemic index score. The question related to consumption of foods high in sugar was correlated with the average daily intake of added sugar. The question related to intake of foods high in fibre was correlated with the servings of whole grains. The question related to spacing carbohydrate throughout the day was correlated with the total carbohydrate spacing score. The question related to eating fish or foods high in *n*-3 fatty acids was correlated with the number of foods in the dietary recall that were high in *n*-3 fatty acids. The question related to using healthy oils was correlated with the intake of monounsaturated fatty acids. The question related to eating foods high in fat was correlated with the intake of total fat. The correlation coefficients were interpreted by using Dancey and Reidy’s categorisation [27].

Reliability: the intra-class correlation coefficient (ICC) was calculated as an indication of test-retest reliability and internal consistency was measured by Cronbach’s α coefficient [28].

## 3. Results

A total of 73 participants were enrolled in the PANDA study, which provided data for validity testing and internal consistency, and 27 participants were separately recruited for test-retest reliability testing. The characteristics of the participants are reported in Table 2. There were no significant differences between demographic characteristics of participants in the first *versus* second cohort except for employment status (*p* = 0.008).

The score for the PDAQ was normally distributed and ranged from 10 to 54. PDAQ scores were not statistically significant different between male (32.5 ± 10.6, *n* = 39) and female (32.9 ± 12.2, *n* = 34) participants. A significant positive correlation was found between PDAQ score and age with r = 0.46, 95% CI (0.19, 0.54), and inversely with weight with *r* = −0.36, 95% CI (−0.52, −0.05).

Total PDAQ scores were associated with nutrient intakes from the average of the three 24-h dietary recall and correlated moderately with HEI-score (*r* = 0.41, 95% CI (0.19, 0.54)), as well as with Vegetables and Fruits servings (*r* = 0.25, 95% CI (0.03, 0.50)). In contrast, total PDAQ scores were negatively correlated with added sugar intake (*r* = −0.32, 95% CI (−0.51, −0.12)) and saturated fat intake (*r* = −0.25, 95% CI (−0.46, −0.05)).

**Table 2:** Baseline characteristics of the first cohort (*n* = 73) and the second cohort (*n* = 27).

Characteristics	Cohort I (*n* = 73)	Cohort II (*n* = 27)	*p*-Value
Age (years)	59.2 ± 9.7	62.8 ± 8.4	0.096
Duration of diabetes (years)	9.1 ± 8.3	11.8 ± 7.8	0.127
Gender, %			
*Male*	53.4%	59.3%	
*Female*	46.6%	40.7%	0.603
Ethnicity, %			
*White*	87.7%	70.3%	0.223
*Other*	12.3%	29.7%	
Education, %			
*High school or less*	15%	7.4%	0.376
*More than high school*	85%	92.6%	
Employment, %			
*Wages and salaries*	56.2%	18.5%	0.008
Household income, %			0.688
*≤$59,999*	21.9%	18.5%	
*≥$60,000*	78.1%	81.5%	

To test the validity, we associated individual items of the PDAQ with nutrient intakes from the average of the three 24-h dietary recalls adjusted for total calories (Table 3). Following CFG more days per week was associated higher intake of servings from a variety of the four food groups (*p* < 0.05). Perceived eating of the recommended servings of Vegetables and Fruits more days per week was associated with higher intake of Vegetables and Fruits reported in 24-h recalls (*p* < 0.05). Reported consumption of foods with a low glycemic index more days per week predicted lower glycemic load (*p* < 0.05). Reporting eating of foods high in sugar (e.g., cookies) on more days was associated with higher added sugar intake (*p* < 0.01). Perceived eating of foods high in fiber (e.g., oatmeal) predicted higher intake of whole grains (*p* < 0.001). Reported consumption of foods high in fat (e.g., fried food) on more days predicted higher fat intake (*p* < 0.01). No significant association was found for spacing carbohydrate, foods high in *n*-3 fatty acids, and healthy oils *versus* the actual intake.

Table 3 presents the correlations between following CFG more days per week (Question (1) of PDAQ) with each subscale (Questions (2)–(9)). Higher perceived adherence to following CFG was moderately correlated with higher intake of Vegetables and Fruits (*r* = 0.60, 95% CI (0.42, 0.73)), higher intake of foods with low glycemic index (*r* = 0.28, 95% CI (0.04, 0.48)), higher intake of foods high in fiber (*r* = 0.44, 95% CI (0.22, 0.61)), more likely to space carbohydrate throughout the day (*r* = 0.59, 95% CI (0.40, 0.75)), and higher intake of fish high in *n*-3 fatty acids (*r* = 0.27, 95% CI (0.08, 0.45)). Conversely, there were negative correlations between perceived adherence of following CFG and intake of foods high in sugar (*r* = −0.36, 95% CI (−0.55, −0.18)) and foods high in fat (*r* = −0.45, 95% CI (−0.63, −024)).

**Table 3:** Validity of Perceived Dietary Adherence Questionnaire (PDAQ) *versus* three 24-h dietary recalls *.

PDAQ Item	PDAQ Score (Mean ± SD) (Maximum 7)	24 h Dietary Recall Item	Intake (Mean ± SD)	Linear Correlation Coefficient Between PDAQ Score and Intake
Following CFG	3.0 ± 2.5	Servings from the four food groups	15.8 ± 3.7	0.33 *
F&V servings	4.1 ± 2.3	F&V servings	4.9 ± 1.9	0.30 *
Low GI	3.6 ± 1.9	Glycemic load	49.5 ± 4.8	−0.30 *
High sugar foods	2.7 ± 2.2	Added sugar (g)	47.4 ± 37.1	0.40 **
High fiber foods	5.0 ± 1.9	Servings of whole grain foods	5.6 ± 2.2	0.46 ***
Carb spacing	3.5 ± 2.6	At least 15 g carbohydrate per meal (maximum 6)	4.3 ± 0.8	0.24
*n*-3 FA	1.7 ± 1.6	*n*-3 PUFA (g)	0.7 ± 2.1	0.11
Healthy oils	3.0 ± 2.5	MUFA (g)	28.7 ± 11.2	0.15
High fat foods	2.6 ± 1.7	Total fat (g)	83.9 ± 30.7	0.35 **

* *N* = 64 participants who completed three 24-h recalls. Abbreviations and explanation: CFG = Eating Well with Canada’s Food Guide; F&V = Fruits and Vegetables; GI = glycemic index; Carb Spacing = Spacing carbohydrate throughout the day; FA = fatty acids; Healthy oils = consumption of foods like nuts, olive oil, canola oil; PUFA = polyunsaturated fatty acids; MUFA = monounsaturated fatty acids. Confidence intervals for significant correlations are reported in the text. * *p* < 0.05; ** *p* < 0.001; *** *p* < 0.0001.

Test and re-test reliability was assessed by the intra-class correlation. High correlations were obtained for five items on the PDAQ (Vegetables and Fruits, foods high in sugar, foods high in fiber, fish and other foods high in *n*-3 fatty acids, and healthy oils) as well as the total PDAQ score (Table 4). Cronbach’s α was 0.78 with no significant change to the overall α with the deletion of any individual item.

**Table 4:** Spearman rank-order correlations between frequency of following Canada’s Food Guide and other items in the Perceived Dietary Adherence Questionnaire (PDAQ).

PDAQ Item	CFG
CFG	--
F&V servings	0.604 **
Low GI	0.280 *
High sugar foods	−0.368 **
High fiber foods	0.414 **
Carb spacing	0.594 **
*n*-3 FA	0.272 *
Healthy oils	0.19
High fat foods	−0.453 **

Abbreviations and explanations: CFG = Eating Well with Canada’s Food Guide; F&V = Fruits and Vegetables; GI = glycemic index; Carb Spacing = Spacing carbohydrate throughout the day; FA = fatty acids; Healthy oils = consumption of foods like nuts, olive oil, canola oil. * *p* < 0.05; ** *p* < 0.001; *** *p* < 0.0001.

## 4. Discussion

The aim of this study was to establish the validity and reliability of a dietary assessment tool for people with type 2 diabetes that would be simple to administer and score, as well as reflect current recommendations for a diabetes diet. Overall, the PDAQ appears to be a useful indicator of adherence to CFG and diet quality. Compared with a repeated 24-h recall, it also appears to be valid for assessing adherence to recommended servings of Vegetables and Fruit, and foods that have low glycemic index, are high in sugar, fiber or fat. The test-retest reliability was acceptable.

Other authors have developed short questionnaires to assess intake of various foods or nutrients in the general population whereas the PDAQ is targeted to specific nutrition recommendations for diabetes. The correlation obtained for Vegetables and Fruits intake between PDAQ and 24-h recall is comparable to previous studies that found moderate correlation (*r* = 0.36–0.65) between Vegetables and Fruit and short food frequency questionnaires [29,30] or seven-day food records [31]. Likewise, other short questionnaires found similar moderate correlations with foods high in sugar, fat and fiber with food records or FFQs [30,31,32]. Poorer correlation was found for foods low in glycemic index in our study compared to other studies, which used short food frequency questionnaires [33,34]. The correlation between self-reported carbohydrate spacing and the carb spacing score derived from 24-h recalls was not significant, which may due to lack of knowledge among diabetic patients [35] as well as health care providers [36], who are thus unable to instruct patients in the technique. No significant relationship was observed between questions related to unsaturated fat and the actual intake of unsaturated fat, which is consistent with Francis and Stevenson´s questionnaire compared with a 4-day food diary [32]. Overall, the PDAQ performed similarly to other short questionnaires and has the advantage of being specific for a particular population, patients with diabetes living in Canada.

We determined that PDAQ had acceptable internal reliability since Cronbach’s α was 0.78 (Cronbach’s α scores for subscales were also acceptable and ranged from 0.74 to 0.79). The test-retest correlation coefficient for the entire questionnaire was acceptable (*r* = 0.76) suggesting that the PDAQ score is stable over time. Test-retest administration of PDAQ produced good correlations for questions related to Vegetables and Fruits, foods high in sugar and fibre, fish or foods high in *n*-3 fatty acids, and healthy oils; meanwhile, questions related to spacing carbohydrate and foods high in fat had moderate correlations (*r* = 0.40 and 0.53, respectively). The question related to CFG had poor test-retest correlation (*r* = 0.21). Low and moderate ICC values in some individual scores are due to the intra-individual variability [21], which is likely to be greater in foods that are consumed less often (like fish in the prairie provinces of Canada). Low test-retest reliability for high fat foods is interesting, suggesting either that there is true variation in intake or that fat may be “hidden” in some foods, such as processed foods [37].

The correlations produced in validity tests between following CFG more days per week and each subscale shows that the PDAQ ranked subjects quite well. We showed that reporting consistent following of CFG is more likely to be positively associated with the intake of low-caloric density foods, and negatively associated with high-caloric density foods. This finding indicates that the PDAQ is a good instrument to measure adherence to CFG recommendations. We were particularly interested in examining PDAQ’s ability to assess intakes specifically mentioned in the CDA Nutrition Therapy Guidelines that may not be captured using scores like the Healthy Eating Index. PDAQ subscales for low GI foods, high sugar, fiber and fat foods were moderate predictors of intake substantiated by the 24-h recall data.

We also found correlations between PDAQ and demographic or biological variables. The positive relationship found between PDAQ and age is similar in direction to a previous study that examined the association between HEI and age [23]. The diet quality of Americans older adults measured using HEI was better than younger and middle-aged adults [38]. PDAQ scores were also significantly negatively correlated with weight, which is consistent with Pate and colleagues’ finding that diet quality was inversely associated with weight status [39]. There was no significant relationship observed between PDAQ and gender.

Several other short questionnaires for dietary assessment have been developed. Calfas and colleagues [9] conducted a review to identify dietary measures that can be potentially used in a primary care setting. All of the instruments measured fat, and some of the instruments measured cholesterol, fruits, vegetables, and fiber. Pullen and Walker [13] used the Behavioral Risk Factor Surveillance Survey to assess adherence to the Dietary Guideline for Americans among midlife and older rural women. The Dutch Diet Index [14] and the Australian Recommended Food Score [40] were developed to measure the adherence to country-specific dietary guidelines. All the previous studies have assessed the reliability and validity of the instruments in the general population. Hemio and colleagues [31] developed a 16-Item Food Intake Questionnaire and used it in a type 2 diabetes prevention programme in Finland to estimate daily nutrient intake in a primary health care setting. To our knowledge, there are no other comparable questionnaires to assess adherence to diabetic recommendations in Canada. Therefore, PDAQ could be a useful tool for dietitians as well as practitioners who are not nutrition experts but who would like a snapshot of the dietary compliance of individuals with type 2 diabetes in Canada. It could also be easily adapted to other settings using the relevant disease and/or country-specific guidelines. In our ongoing research we are using PDAQ to assess longitudinal changes in dietary adherence in type 2 diabetes participants. Preliminary analyses suggest that PDAQ is useful for this purpose [41].

One strength of our study is that we used three internet-based, 24-h dietary recalls to estimate dietary intake, a method that has less bias than some others [20] and was also relatively simple for the participants to complete. The study developed a short, simple to administer and score questionnaire that covers the CDA Nutrition Therapy guidelines [18] with reference to following CFG [8]. Use of PDAQ could therefore reduce both client and practitioner burden but allow longitudinal monitoring of dietary adherence to recommendations. The study has some limitations that need to be recognised. This study has a relatively small sample size but some previous studies have validated dietary instruments with a similar number of participants [31,32,39]. However, the small sample size does limit our ability to conduct multivariate or subgroup analyses such as gender or age effects. All participants lived in an urban area, therefore, the result may not be generalizable to those living in rural areas. Another limitation is that participants in the intervention study were more educated and had higher income compared with the general population. Although this may not affect the validation study, our findings might be different if we apply it a population with lower education and income. Finally, although the CDA does not have a specific recommendation for sodium, our studies such as that reported in [41] consistently find sodium intake in excess of current Health Canada guidelines [42]. An item related to sodium intake could be a useful addition to the PDAQ.

## 5. Conclusions

Following the CDA nutrition therapy guidelines is important for improving health outcomes in people with type 2 diabetes, but there is a need to develop practical and quick tools that help clinicians and researchers to assess adherence to these guidelines. We suggest that the PDAQ may be useful to accomplish this objective and that it can be implemented in research. It may be worthwhile to test the PDAQ in a clinical setting.
